# Diabetes Insipidus after Traumatic Brain Injury

**DOI:** 10.3390/jcm4071448

**Published:** 2015-07-13

**Authors:** Cristina Capatina, Alessandro Paluzzi, Rosalid Mitchell, Niki Karavitaki

**Affiliations:** 1 Carol Davila University of Medicine and Pharmacy, Endocrinology Department, 34–36 Aviatorilor Blvd, Bucharest 050474, Romania; E-Mail: cristina.capatina@yahoo.com; 2 Neurosurgery, University Hospitals Birmingham NHS Foundation Trust, Queen Elizabeth Hospital, Birmingham, B15 2TH, UK; E-Mails: Alessandro.Paluzzi@uhb.nhs.uk (A.P.); Rosalid.Mitchell@uhb.nhs.uk (R.M.); 3 Centre for Endocrinology, Diabetes, and Metabolism, Institute for Biomedical Research, School of Clinical and Experimental Medicine, University of Birmingham, Birmingham B15 2TT, UK

**Keywords:** traumatic brain injury, diabetes insipidus, hypernatremia, polyuria

## Abstract

Traumatic brain injury (TBI) is a significant cause of morbidity and mortality in many age groups. Neuroendocrine dysfunction has been recognized as a consequence of TBI and consists of both anterior and posterior pituitary insufficiency; water and electrolyte abnormalities (diabetes insipidus (DI) and the syndrome of inappropriate antidiuretic hormone secretion (SIADH)) are amongst the most challenging sequelae. The acute head trauma can lead (directly or indirectly) to dysfunction of the hypothalamic neurons secreting antidiuretic hormone (ADH) or of the posterior pituitary gland causing post-traumatic DI (PTDI). PTDI is usually diagnosed in the first days after the trauma presenting with hypotonic polyuria. Frequently, the poor general status of most patients prevents adequate fluid intake to compensate the losses and severe dehydration and hypernatremia occur. Management consists of careful monitoring of fluid balance and hormonal replacement. PTDI is associated with high mortality, particularly when presenting very early following the injury. In many surviving patients, the PTDI is transient, lasting a few days to a few weeks and in a minority of cases, it is permanent requiring management similar to that offered to patients with non-traumatic central DI.

## 1. Introduction

Diabetes insipidus (DI) is caused by decreased secretion (central/neurogenic DI) or action (nephrogenic DI) of antidiuretic hormone (ADH, vasopressin). ADH is produced by the hypothalamic neurons in the supraoptic and paraventricular nuclei, migrates along their axons to the posterior pituitary gland where it is stored in secretory granules and is secreted in the circulation when stimulated (by increased plasma osmolality—osmoregulation or by decreased arterial blood pressure—baroregulation). ADH acts on specific receptors (vasopressin receptors; three subtypes V1a, V1b and V2 have been identified). Its main physiological effect (increase of water absorption in the distal nephron) requires the stimulation of local V2 receptors promoting the expression of specific water channel proteins (aquaporins) on the luminal surface of the collecting duct [1].

DI manifests with loss of large volumes of dilute urine in the presence of normal or (usually) high plasma osmolality. Central DI is the result of a number of conditions affecting the hypothalamic-posterior pituitary unit—see box nr. 1.

○ Neurosurgical interventions
○ Traumatic brain injury (TBI)
○ (Para)sellar tumors (e.g., craniopharyngioma, germinoma, meningioma, germ cell tumor)
○ Metastases to pituitary gland (especially from breast/lung malignancies)
○ Infections (e.g., meningitis, abscess, encephalitis)
○ Infiltrative diseases (e.g., sarcoidosis, histiocytosis, lymphocytic hypophysitis)
○ Vascular abnormalities (aneurysm)
○ Autoimmune
○ Genetic (AD, AR, X-linked recessive, DIDMOAD)
○ Congenital (e.g., septo-optic dysplasia, holoprosencephaly, midline defects)

box nr. 1. Causes of central diabetes insipidus—data from [2,3]; AD-autosomal dominant; AR-autosomal recessive; DIDMOAD (Diabetes Insipidus, Diabetes Mellitus, Optic Atrophy, and Deafness) (AR transmission).

## 2. Pathophysiology of Posttraumatic DI (PTDI)

Traumatic brain injury (TBI) is a significant public health problem. It is associated with high mortality, as well as acute and chronic morbidity with many survivors being severely disabled physically and psychologically [4,5,6]. It is more frequent in children, young adults and the elderly [5] and it is the leading cause of death in young individuals [7].

TBI is mainly the result of road traffic accidents, assaults, falls and domestic abuse [8]. The pathogenic event leading to dysfunction of the hypothalamic-pituitary axis is attributed to the direct mechanical impact, to the acceleration-deceleration effect in motor vehicle accidents or to the cerebral consequences of trauma (ischemia, hypoxia, alterations of cerebral vascularization or metabolism, increased intracranial pressure). Pituitary damage secondary to TBI was reported as early as 1918 by Cyran. [9] Later studies by Holborn [10] suggested that changes in the rotational velocity of the head (with subsequent stretching or tearing of small vessels or neuronal structures) are the main mechanisms of the posttraumatic damage of the hypothalamo-pituitary unit. These mechanisms have been also proposed by Porter *et al.*, in 1948 [11].

The damage of the hypothalamic ADH-producing neurons, their axons or the posterior pituitary leads to post-traumatic central DI [12].

PTDI is usually diagnosed after a latent interval and is frequently transient (see natural history below); in cases of transient DI, an indirect pathogenic mechanism (small vessels damage, inflammatory edema) appears more likely than direct neuronal damage [13].

Furthermore, the concomitant damage of the thirst osmoreceptors in the hypothalamus may result in abnormalities in the thirst regulation manifested as either adipsic PTDI (characterized by failure of high plasma osmolality to stimulate ADH secretion and thirst and associated with severe hypernatremia and increased mortality [14]) or, rarely, as PTDI associated with primary polydipsia [15]. It should be noted however, that concomitant injury of the thirst center is very rare in this setting, as opposed to other pathologies where adipsic DI is more extensively reported (e.g., after craniopharyngioma surgery [16]).

In autopsy studies of patients not surviving severe head trauma [17], stalk hemorrhage or infarction, hemorrhages in the hypothalamic nuclei or the infundibular region have been described [18].

The pattern of the endocrine abnormalities following brain trauma vary depending on the site of the injury. Injury predominantly to the hypothalamus can cause anterior hypopituitarism, PTDI or inappropriate secretion of antidiuretic hormone (SIADH) [13]. Damage to the posterior pituitary only does not usually lead to permanent DI as ADH produced in the hypothalamic nuclei can still be found in the peripheral circulation via the capillaries of the median eminence [18]. After pituitary stalk transection, a triphasic response has been described (initially DI, followed a few days later by transient SIADH and later recurrence of DI, either transient or permanent). This is attributed to the shock of the initial injury, followed by the release of pre-synthesized ADH and then a recurrence of ADH deficiency due to impaired synthesis of the hormone by the damaged neuronal structures. This classical presentation is rarely seen in the clinical practice [19].

## 3. Epidemiology of PTDI

The pituitary gland, its vascular supply and the stalk are highly susceptible to trauma [17] and TBI has been frequently associated with anterior and/or posterior pituitary dysfunction [20,21].

TBI is commonly associated with abnormalities of the water and sodium balance (SIADH and DI) [22]. Their timely diagnosis is of major importance as, if unrecognized, they can lead to severe electrolyte disturbances [23].

The epidemiological data on PTDI are affected by a number of inconsistencies amongst the published studies: heterogeneity of the diagnostic criteria used, of the characteristics of the studied population (different degrees of TBI severity) and of the timing of evaluation. As a consequence, the true prevalence of PTDI is difficult to be estimated, ranging between 2.9%–51% [8,24]. In two series by Agha *et al.*, 21.6%–26% of the patients developed PTDI in the acute setting [25]. Hadjizacharia *et al.*, reported that 15% of the TBI cases were diagnosed with PTDI, mostly in the first few days after the injury (mean 1.2 days) [26]. In a prospective study of severe TBI patients, 28% developed DI [27]. Furthermore, in a series by Benvenga *et al.*, 20% of the post-TBI hypopituitary patients also developed transient DI [28]. The same group reviewed the literature and reported that 30.6% of the cases with post-traumatic hypopituitarism also had PTDI [28]. On the other hand, Boughey *et al.*, found prevalence of 2.9% but in their series only severe cases were included (with mean plasma sodium concentration 161 mEq/L) [8]. Finally, in other studies the prevalence of acute PTDI is notably higher (50%–51%) [24,29].

Risk factors for PTDI include low Glasgow coma scale (GCS) score, cerebral edema and severe injury [25,29]. Although acute DI is generally associated with more severe TBI [30], it can also occur in cases of mild head injury [31].

## 4. Natural History

Studies looking at the natural history of PTDI are scarce with methodological limitations related to the testing protocols and the diagnostic criteria used. In many reports the criteria proposed by Seckl and Dunger are followed (polyuria >3l, urine osmolality below 300 mosm/kg, hypernatremia >145 mmol/L) [32].

PTDI is frequently a transient condition. Agha *et al.*, reported an overall prevalence of persistent DI of 6.9% at 6–36 months after the injury (the patients were assessed using the standard water deprivation test (WDT)) [25]. In another prospective study from the same group involving 50 consecutive TBI patients evaluated by a WDT at 6 and 12 months after the trauma, the results were very similar: out of the 13 cases (26%) initially diagnosed with PTDI, 9 recovered in the first 6 months and 10 in the first year, whereas persistent PTDI was found in only 6% of all TBI cases [30]. The recovery is possibly attributed to the slow involution of the edema and the vessel regeneration in the affected areas. This may explain the higher percentage of persistent DI in studies assessing the patients soon after the traumatic event. Thus, in one study evaluating subjects with mild traumatic injury five weeks after the event, 21% were diagnosed with persistent DI [33]. Apart from the short time interval, this figure was probably an overestimate also due to the non-strict diagnostic criteria used (hypotonic urine defined as having an osmolality <1000 mosm/kg) [33]. In another series, at three months after the injury, 4.2% of the cases had persistent PTDI, whereas at 1 year, the percentage decreased to 2.8% [34]. The corresponding figures in an adolescent population investigated by the same group at the same time intervals were 8.6% and 4.3%, respectively [35]. Notably, in many cases, full recovery of transient DI occurs during the initial admission: 40 out of 51 acute PTDI cases recovered during the hospital stay in a large study of moderate-to severe TBI cases [24].

The diagnostic methods used have a significant impact on the reported rates of persistent DI. In a mixed series of previously brain-injured cases (mostly traumatic, 80%), none of the 70 patients assessed at a median time interval of 13 months after the episode had persistent DI. Since these were mostly severely injured cases, there is no obvious explanation for these findings. It is, however, of note that no details were provided on the diagnostic tests and criteria used or on the prevalence of PTDI in the acute phase [36]. The WDT is likely to diagnose mild, partial DI cases leading to more accurate prevalence rates of chronic PTDI (between 2% [37] and 7% [25]).

## 5. Clinical Manifestations

Patients with DI lose their ability to concentrate urine and pass large volumes of dilute urine (polyuria) consequently experiencing polydipsia. In mild PTDI cases with preserved consciousness and thirst sensation, these will prompt thorough investigation. However, most of the PTDI patients are in a poor status during admission in the intensive care unit and their ability to express the feeling of thirst or to drink is impaired. In these cases, high volume inappropriate dilute urine and hypernatremia are key points for the diagnosis. The initial assessment of TBI patients is based on the Glasgow Coma scale (GCS) score [38] and severe injury (defined by a score ≤8 [39]) is commonly found (in one series, 88% of the patients had GCS score ≤6) [8]. In such severely affected subjects, impaired consciousness (due to the direct traumatic effect, cerebral edema, intracranial hemorrhage or the sedative medications used in the intensive care unit [40]), impaired thirst sensation, inability to consume fluids (e.g., due to associated lesions in the oropharyngeal area) frequently occur. The inability to consume fluids and compensate the renal water losses rapidly results in severe dehydration and hypovolemic hypernatremia (with hypotension and low cerebral perfusion pressure). The signs of dehydration (decreased skin turgor, dry mucous membranes, hypotension, tachypnea, tachycardia, confusion, hypovolemic shock, renal failure) need to be constantly monitored and interpreted in relation with the adequacy of fluid replacement. Furthermore, given that the neurological signs of hypernatremia (confusion, disorientation, hyperreflexia, seizures, lethargy, coma) [41,42,43] are difficult to be differentiated from other causes of altered neurological status, the monitoring should always be supported by regular assessment of fluid input and output and of plasma electrolytes concentration (especially sodium).

The presentation of PTDI occurs most frequently in the first few days following the trauma: 2–3 [30] to 4–10 [24,26]. Generally, no new cases of DI are diagnosed after the acute phase [25], although exceptions have been described [44,45]. Extremely rapid onset of DI symptoms (first hour after the injury) has also been reported [46]. Development of DI in the first day after injury has been associated with very high mortality [8] but very early onset of DI followed by complete remission has also been exceptionally described [46]. Overall, PTDI is diagnosed significantly earlier in patients who do not survive following the TBI [8].

## 6. Diagnosis

The diagnosis of PTDI is not always straightforward, particularly in the intensive care setting where patents may require treatment with hyperosmolar substances or use of barbiturates (which are delivered with sodium) and careful volume regulation aiming to prevent further increase in brain edema. Moreover, polytraumatized patients often have high blood loss and need volume replacement which may interfere with the diagnosis of DI.

As soon as polyuria is detected, exclusion of other causes of increased urinary fluid losses is required. This includes hyperglycemia (frequent in trauma patients as a result of the hypercatabolic state and medications), administration of hyperosmolar fluids (e.g., mannitol or hypertonic saline), diuretics, excessive fluid replacement, urea diuresis (excess of urea from tissue hypercatabolism) [41]. In these cases, the solute diuresis results in increased urine osmolality, as opposed to the dilute urine found in the water diuresis of DI.

In all TBI patients passing increased volumes of hypotonic urine, PTDI should be considered and the blood levels of electrolytes (especially sodium, but also potassium and calcium, as hypokalemia and hypercalcemia are also associated with polyuria) with simultaneous plasma and urine osmolalities need to be checked. In patients without cognitive impairment and preserved ability to drink, the sodium concentration may remain normal as the increased oral fluid intake can match the high urine output [47]. In these cases, the diagnosis should be confirmed in the post-acute phase by the standard WDT [30]. The confirmation of DI in hypernatremic cases relies on the demonstration of low urine osmolality in the presence of plasma hyperosmolality. It should be noted however, that the diagnostic criteria for PTDI are not clearly established. Thus, polyuria has been defined as urine output >30 mL/kg body weight or >200 mL/h for 2 h consecutively [8,27] or >5 mL/kg/h [48]. In other series [24,27,49], the criteria proposed by Seckl and Dunger are applied; polyuria (>3 L/24 h) with hypotonic urine (urine osmolality <300 mosm/kg) and plasma sodium concentration >145 mmol/L reliably diagnose acute DI [32]. Notably, Agha *et al.*, diagnosed PTDI based on the combination of polyuria (>3.5 l/24 h) with dilute urine (urine/plasma osmolality <2), hypernatremia (>145 mmol/L) and increased plasma osmolality (>300 mosm/kg) [30].

ADH measurements do not seem to provide significant benefit as overlap occurs between various diagnoses [50] and the post-traumatic state is an additional source of potential confounding factors (e.g., hypotension, emesis, concurrent adrenal insufficiency). Additionally, in patients in the intensive care, the ADH secretion is stimulated resulting in higher serum ADH concentrations compared to controls [51].

The concentration of copeptin, the C-terminal glycopeptide of the ADH prohormone (found to be significantly lower in central DI cases compared to normal subjects) [52] has not been evaluated in post-traumatic DI cases. Notably, in the critically ill patients, serum copeptin concentrations are high [51] and significantly correlated with the severity of the injury [53].

Interestingly, DI and cerebral salt wasting syndrome (CSW) can occur sequentially in the same individual [54], most frequently in CNS infections but also after head trauma [48].

Patients diagnosed with PTDI should be also checked for anterior pituitary dysfunction both in the acute phase and during follow-up.

## 7. Imaging

Brain imaging (CT or MRI) is routinely performed in TBI patients. Intracranial hemorrhage (intracerebral, subarachnoid, subdural) and cerebral edema are very frequently demonstrated [8]. Skull fractures, cerebral contusions, subdural or epidural hematoma can also be found [55]. CT is as useful as MRI in detecting intracranial hemorrhage, subarachnoid hemorrhage (SAH), hematomas, and cerebral contusions and more sensitive in revealing skull fractures [56].

Overall, the imaging findings can be classified as showing focal brain injury or diffuse injury. Focal injury of the hypothalamic-pituitary region can be demonstrated on cerebral MRI images in 30% of all TBI cases: focal pituitary changes (hemorrhage or infarction), increased gland volume and stalk transection [57]. Pituitary stalk hematoma [58] or loss of the bright signal intensity of the pituitary posterior lobe on T1-weighted scans [29] are occasionally seen. An ectopic bright spot in cases with stalk transection has been rarely described [59].

Occasionally (around 6% of cases), cerebral CT/MRI fail to demonstrate any abnormal finding. In these cases, either hypoxic damage or diffuse axonal injury are presumably responsible [28].

## 8. Management

The initial intensive care management of TBI cases follows established protocols with close monitoring of parameters including cerebral perfusion pressure (CPP), intracranial pressure (ICP) and the oxygenation status [40]. Sedatives are routinely administered [40]. Furthermore, clinical and standard laboratory assessments remain essential. Skin turgor, mucous membranes hydration status, heart rate, blood pressure, GCS score should be constantly monitored. An indwelling urinary catheter to evaluate reliably the urine output is mandatory. Fluid intake, urine specific gravity (SG), plasma and urine osmolalities, plasma sodium should be checked frequently [60].

**Figure 1:**
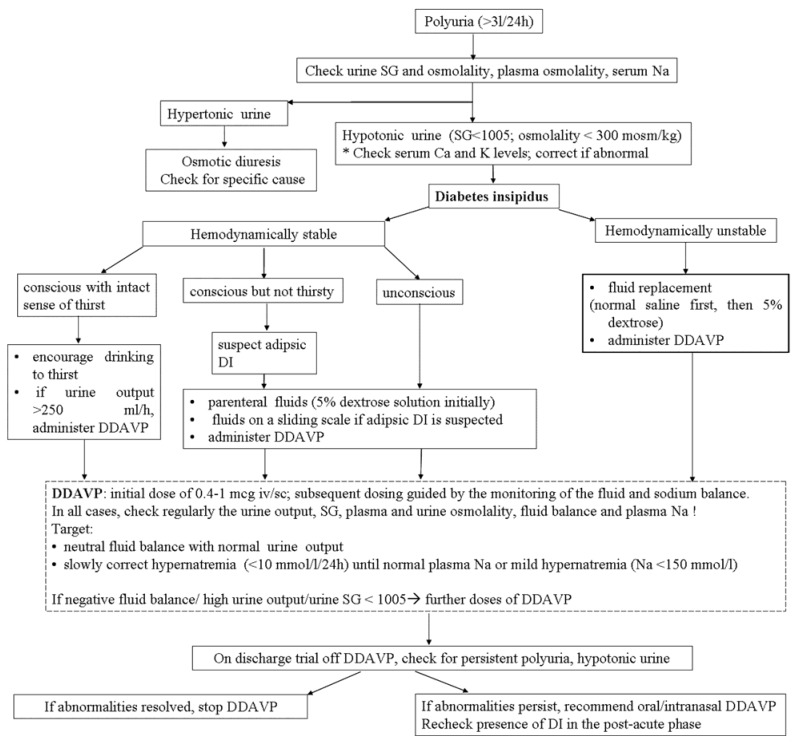
Proposed algorithm for the diagnosis and management of PTDI *iv-*ntravenous; *sc*-subcutaneous.

After the diagnosis of DI, the initial approach aims to replace fluids in order to avoid dehydration (which is associated with an adverse outcome in acute head-injured patients) [61]. If the patient is conscious, has normal thirst sensation and the general physical status allows it, the oral fluid intake usually compensates the renal water loss and the fluid balance is preserved with no dehydration or hypernatremia. In these patients, conservative management and frequent monitoring of fluid and electrolyte balance, as well as of plasma/urine osmolalities is offered [62], with hormone replacement treatment reserved for those with urine output >250 mL/h [63]—see Figure 1.

In cases with altered consciousness, associated neurological deficits or dysphagia and possible alterations of the thirst mechanism, hypotonic polyuria can rapidly lead to hypovolemia and hypernatremia. Hypovolemia should be initially corrected by intravenous administration of fluids and accurate assessment of the volume status is mandatory [23]. 5% dextrose solution is preffered for fluid replacement with isotonic saline reserved exclusively for the hemodynamically compromised cases [41,64].

Fluid replacement should be guided by constant clinical monitoring and CVP measurements to avoid both under-replacement (associated with hypovolemia and decreased CPP) and over-replacement (which aggravates cerebral edema, increases the ICP and can precipitate pulmonary edema). Fluid replacement measures will also decrease plasma sodium and may be adequate to correct mild hypernatremia (<150 mmol/L). However, in severe cases, the plasma sodium can be high [48] and this strongly relates to increased mortality [65,66]; hypernatremia should be slowly corrected as the cerebral tissue is hypersensitive to quick osmotic changes leading to worsening of the cerebral edema. A correction rate of no more than 0.5 mmol/h [67] or 10–12 mmol/L/24 h [42,43,44] is recommended. A suggested practical approach is to decrease plasma sodium levels by 1 mmol/L/h in the first hours and then decrease the rate aiming to a reduction of no more than 12 mmol/L per 24 h [44]. Furthermore, hormonal replacement with desmopressin is also needed.

Native vasopressin injections have a less favorable side-effect profile due to concomitant stimulation of V1 receptors, as well as to the possible development of anti-vasopressin antibodies (associated with a subsequent lack of response to treatment) [66]. Although vasopressin could have an advantage in the acute setting due to the lower duration of action and option of more frequent dose adjustment, the above considerations and the low availability worldwide have limited its use. DDAVP (deamino-arginine-vasopressin, desmopressin)—a synthetic analog of vasopressin with minimal pressor effects is currently the drug of choice for the management of central DI [68]. Desmopressin can be administered orally, intranasally, sublingually or parenterally. Parenteral desmopressin formulations are preferred in the acute intensive care setting where fluid intake is determined by factors other than thirst but there are no guidelines on the optimal protocol. Initially, a single dose is administered [24] (0.4–1 micrograms intravenously or subcutaneously [69,70]).With parenteral administration of desmopressin, the usual duration of action is 12 h [71]; however, significant inter-individual variability occurs in the duration and the amplitude of the effect [72] and treatment with additional doses should be dictated by careful assessment of the fluid and electrolyte balance. In order to avoid the risks of over-treatment, the minimum dose of desmopressin required to normalize urine output and provide the required rate of plasma sodium reduction is recommended. Each extra dose of desmopressin should be offered only if there is evidence of persisting abnormalities in the fluid balance, SG and plasma sodium.

Caution is also required for the later development of traumatic cerebral salt wasting syndrome (CSW) which predisposes to rapid hyponatremia [48,54]. However, this has been very rarely reported. Furthermore, careful monitoring of the fluid and sodium balance is mandatory for the recognition of the occasional triphasic response.

Concomitant ACTH deficiency should always be considered and checked for; in such a case, the DI appears milder or improving due to the decreased renal ability to dilute the urine [73].

In adipsic PTDI, fixed desmopressin doses are recommended together with fluid replacement in a rate dictated by the plasma sodium concentration and osmolalities [49,74]. In these patients, prophylaxis against thromboembolism is also indicated [49] as thromboembolic complications may develop due to severe dehydration [15].

On discharge, treatment withdrawal should be attempted to confirm persistence of the DI; in this is the case, oral or intranasal desmopressin formulations are recommended. The oral desmopressin has low bioavailability but good antidiuretic effect [3] and the ease of administration makes it a preferred option for chronic treatment. A usual maintenance dose is about 100 to 200 micrograms three times daily but requirements vary; therefore, lower doses are advised initially and further increase is individualized aiming to maintain normal urine output and plasma sodium. A sublingual lyophilisate (melt) formulation of desmopressin with better bioavailability [75] is also available (in 60, 120, and 240 micrograms form). Long-term dosage should follow the same protocol as in the oral preparations. Intranasal formulations are offered usually in doses of 10–40 micrograms daily (divided once or twice daily) (significantly lower than the oral ones because of improved bioavailability associated with the lack of action of gastrointestinal peptidases). In clinical studies, intranasal and oral administration have similar efficacy [76].

The goal of chronic treatment is to control the polyuria and the electrolyte disturbances without inducing hyponatremia (or water intoxication); this may require adjustment of the dose of desmopressin and the patient should be clearly instructed to adjust the fluid intake to the thirst sensation. It is often useful to allow intermittent polyuric episodes every one to two weeks by delaying a dose of desmopressin; this will verify the continued presence of diabetes insipidus and allow excretion of any retained excess water [3].

Re-evaluation of PTDI cases after the acute phase includes assessment of polyuria, polydipsia and formal WDT (after omitting desmopressin) [30]. If persistent DI is diagnosed, treatment should be continued with formal assessment repeated one year later as a minority of cases may recover even after a long interval from the trauma [30].

Long-term follow up of the anterior pituitary function is also needed as late evidence of traumatic hypopituitarism has been reported [34] and is often underdiagnosed in milder cases [77].

## 9. Prognosis

PTDI has been consistently linked to more severe trauma, cerebral edema, lower GCS scores and a higher mortality rate [26,30]. The occurrence of DI has been associated with brain death as it is present in 80% of the brain-dead patients [78].

The overall mortality of TBI patients with PTDI ranges between 57%–69% and increases to 86%–90% in those with early-onset of DI, in the first three days from injury [8,27].

Peak plasma sodium concentration is also of prognostic value [47] with significantly higher values in non-survivors [8,27]. Notably, in one series all cases with a maximum recorded plasma sodium >160 did not survive [48].

The correlation between the severity of trauma and that of chronic persistent endocrine dysfunction is less clear [34]. In contrast to post-surgical cases in which an elevated plasma sodium level in the first five post-operative days can predict an increased risk of developing permanent DI [79], no predictors of long-term persistent PTDI have been identified.

## 10. Conclusions

PTDI occurs mainly after severe head trauma and predisposes to hypovolemia and hypernatremia with significant deleterious consequences for the already severe state of most TBI patients. Careful continuous monitoring in the acute intensive care setting is essential for the prompt diagnosis and optimal management of PTDI aiming at maintaining the fluid and electrolyte balance and decreasing the associated morbidity and mortality.
